# Multiple Cancellous Exostoses

**Published:** 1892-01-02

**Authors:** 


### SURGERY.

### MULTIPLE CANCELLOUS EXOSTOSES.

The aggregate occurrence of exostoses in all varieties is very considerable, and every practitioner of experience must have seen the condition in one or other of its manifestations. The present article deals more particularly with those examples of this interesting abnormality exhibiting the exostoses in multiple number in the same individual, and special reference is here made to six exceedingly well-marked cases which have been the subjects of our recent observation. These cases represent two generations of the same family. A father and his sister; the father's daughter and his three sons, these being all the children of the family.

Whilst heredity is very frequently quoted as a concurrence, we cannot from the recorded cases deduce any definite conclusions as to the general or local etiology nor the particular diathetic attitude or attitudes which are most prone to favour the formation of these multiple exostoses. Payne suggested their probable growth from the residues of embryonic cartilage left in the formation of the bone, and becoming ossified in process of growth, and held that this is supported by the two facts, that the exostoses occur in positions analgoous to those selected by cartilaginous tumours, and that chondromata and exostoses may occur together. These facts would, at least, suggest that these two varieties of tumours are the product of one and the same structural tendency. But he adds that they occur at the sites usually selected for the exhibition of rickets; thus apparently, favouring an association of the ricketty condition with that of multple exostosis. In our example cases, evidences of rickets are undoubtedly present.

Rheumatism, syphilis, inter-marriage, have each been recorded either as possible coincidences or concurrent associations.

The use of the term multiple is often amply justified; the total number, for instance, of palpable exostoses in the six cases specially referred to being four hundred and fifty-four, making an individual average of nearly seventy-six. The disposition of the tumours is often to a marked degree symmetrical. Most frequently they are found in the neighbourhood of the epiphyses of the long bones, though by no means exclusively so; being not rarely found on their shafts and at the synchondroses of rib and cartilage, and in the course of the rib bodies. The crests of the haunch bones may be studded as in three of our six cases, and here it is of interest to note that no definite relation has been made out between this condition and the large intrapelvic exostoses which may prove so troublesome in parturition.

Whilst the scapulæ may be extensively affected, the sternum and patellæ, with face and skull bones, spinal column, tarsal and carpal bones are comparatively exempt. In these six cases the tibia and scapula are most affected; next to these comes the humerus, then the femur and fibula. Excluding the hand bones and scapula from the upper extremity and the foot bones with os innominatum from the lower, the latter is almost twice as much affected. Apart from these exclusions the order is more than reversed.

Many of the exostoses of this variety, situated towards the ends of the long bones, have a very definite relation to the tendinous insertion of the muscles (especially the more powerful muscles), rather than to the proximate epiphysis. They may grow into the tendons for a considerable distance, and may even become separated from the bone. The growth is more exuberant at those ends of the long bones, whose epiphyses unite later, and away from which the nutrient artery is directed.

A tendency to the formation of a bony "anklet" at the lower ends of the leg bones is often present, and is very well marked in one of the boys who, on this account, cannot closely lace the boot. Palpation of this anklet gives the impression that the lower ends of the tibia and fibula are welded together by a bony mass. In a case, however, examined by Virchow, it was found due to flattening against each other of the much and irregularly enlarged lower ends of these bones.

Multiplicity appears early in life, occurring in all recorded cases between infant life and 20 years. They may increase slowly in size till about the 20th year. After this they usually cease to grow, and remain passive for the remainder of life, or they may become appreciably smaller.

Treves remarks that when in the region of joints they may, though rarely, interfere with the range of joint movement. It is remarkable how large an exostosis may be tolerated without any apparent interference with the function of the part affected. The youngest of the three boys has a very large one, felt in the right upper calf, causing a tumour quite the size of a large orange, springing from the posterior surfaces of the heads and upper third of posterior surfaces of shafts of tibia and fibula. This, of course, limits extreme flexion of the leg or the thigh, but no evidences of nerve pressure, circulatory interference, or muscular impairment are detectable.

When in positions where they are affected by surface friction, as in the region of the ankles, where the boots rub in locomotion, bursæ may be developed over the tumours, and at times such bursæ become inflamed, causing some inconvenience. When near joints, also, they are not unfrequently covered by a projection of the joint-synovial membrane, and this is a noteworthy fact when removal of exostoses in this site is contemplated.

The structure of these multiple exostoses is well known, being mainly cancellous, usually easily cut, amply porous on section, and capped by a thin coating of cartilage covered by periosteum.

The *diagnosis* is usually made sufficiently clear by their position, hard bony feel, immobility, and slow growth. They often cause aching and pain in the limb.

With regard to *treatment*, all authorities seem satisfied that nothing operative should be proceeded to unless for some special reason, such as severe pain, visible deformity or pressure on important parts. Treves remarks that suppuration often follows their removal, and may track up and down the limb. It may also be added that recurrence at or near the original site, with increased exuberance of growth, is not uncommon.

